# Effects of Experimental *Sarcocystis neurona*-Induced Infection on Immunity in an Equine Model

**DOI:** 10.1155/2014/239495

**Published:** 2014-11-12

**Authors:** S. Rochelle Lewis, Siobhan P. Ellison, John J. Dascanio, David S. Lindsay, Robert M. Gogal, Stephen R. Werre, Naveen Surendran, Meghan E. Breen, Bettina M. Heid, Frank M. Andrews, Virginia A. Buechner-Maxwell, Sharon G. Witonsky

**Affiliations:** ^1^Department of Large Animal Clinical Sciences, Virginia-Maryland Regional College of Veterinary Medicine, Blacksburg, VA 24061, USA; ^2^Rangiora Veterinary Centre, Rangiora 7400, New Zealand; ^3^Pathogenes Inc, P.O. Box 970, Fairfield, FL 32634, USA; ^4^College of Veterinary Medicine, Lincoln Memorial University, Harrogate, TN 37752, USA; ^5^Department of Biomedical Sciences and Pathobiology, Virginia-Maryland Regional College of Veterinary Medicine, Blacksburg, VA 24061, USA; ^6^Department of Biosciences and Diagnostic Imaging, College of Veterinary Medicine, University of Georgia, Athens, GA 30602, USA; ^7^Rochester General Hospital Research Institute, Rochester, NY 14621, USA; ^8^Natural Vet Palm Beach, Juno Beach, FL 33408, USA; ^9^Department of Large Animal Clinical Sciences, College of Veterinary Medicine, University of Tennessee, Knoxville, TN 37996, USA; ^10^School of Veterinary Medicine, Veterinary Teaching Hospital and Clinics, Louisiana State University, Baton Rouge, LA 70803, USA

## Abstract

*Sarcocystis neurona* is the most common cause of Equine Protozoal Myeloencephalitis (EPM), affecting 0.5–1% horses in the United States during their lifetimes. The objective of this study was to evaluate the equine immune responses in an experimentally induced *Sarcocystis neurona* infection model. Neurologic parameters were recorded prior to and throughout the 70-day study by blinded investigators. Recombinant SnSAG1 ELISA for serum and CSF were used to confirm and track disease progression. All experimentally infected horses displayed neurologic signs after infection. Neutrophils, monocytes, and lymphocytes from infected horses displayed significantly delayed apoptosis at some time points. Cell proliferation was significantly increased in *S. neurona*-infected horses when stimulated nonspecifically with PMA/I but significantly decreased when stimulated with *S. neurona* compared to controls. Collectively, our results suggest that horses experimentally infected with *S. neurona* manifest impaired antigen specific response to *S. neurona*, which could be a function of altered antigen presentation, lack of antigen recognition, or both.

## 1. Introduction 

Equine Protozoal Myeloencephalitis (EPM) represents the most commonly diagnosed neurologic disease of horses within the United States [1–3]. Horses are aberrant hosts and are commonly infected by ingestion of the* Sarcocystis neurona* sporocyst, through contamination of feedstuffs. The majority of horses exposed to this parasite generate a protective immune response and do not develop clinical signs. However, for the small percentage of horses that develop EPM, the clinical signs are classically those associated with asymmetric neurologic deficits, including gait abnormalities, ataxia, weakness, and focal muscle atrophy [3–5]. Annual losses in the United States are estimated to be $55.4 to $110.8 million [2].

In order to develop more efficacious treatments, vaccines, and diagnostic assays, it is important to first determine if there is an immune signature that dictates whether a horse will develop a protective response or neurologic disease. While protection has been linked to CD4 and CD8 cell-mediated response and interferon-gamma (IFN*γ*) production [6–9], the specific pathway(s) of immune cell responses associated with development of disease in the equine are still poorly characterized. Interestingly, a number of studies have reported an immune profile of decreased CD4 expression, surface antigen 1- (SAG1-) induced cell proliferation, PMA/I stimulated cell proliferation, and IFN*γ* in EPM affected horses [10–13]. It has been proposed that stress-induced immune dysregulation increases disease susceptibility, as horses subjected to stressful conditions (i.e., shipping, showing, training, and pregnancy) tend to have an increased incidence of disease [14–17]. Therefore, new studies designed to explore the specific immune consequences of EPM in the horse would facilitate new drug discoveries and diagnostic tests which would have great economic and emotional impact for both the individual horse owner and the US equine industry.

Previously, we determined that both naturally and experimentally infected horses have suppressed leukocyte proliferation responses when stimulated with combined mitogens PMA/I [12, 13]. The purpose of the present study was to determine whether this defect was due to an increase in apoptosis or decrease in cellular proliferation of a specific subset of leukocytes, as well as to identify altered immune responses during disease progression in horses experimentally challenged with* S. neurona.*

## 2. Materials and Methods

### 2.1. Horses

Nine-quarter horse and quarter horse crosses, ranging in age from 18 months to 11 years, consisting of eight mares and one gelding, were used in this study. All horses were vaccinated, prior to transport to Virginia Tech, against equine influenza, tetanus, West Nile Virus, Eastern and Western Equine Encephalitis, and Equine Rhinopneumonitis. Upon arrival, the horses were also vaccinated against rabies. Historic records indicated that none of the horses had exhibited clinical signs of neurological disease. Horses were maintained on pasture and fed grass hay and pelleted “sweet feed” in accordance with normal energy requirements for weight gain for the study duration. Horses were acclimated for two weeks prior to assessment of baseline immune function and serology.

### 2.2. Physical and Neurologic Examinations

**Figure 1:**
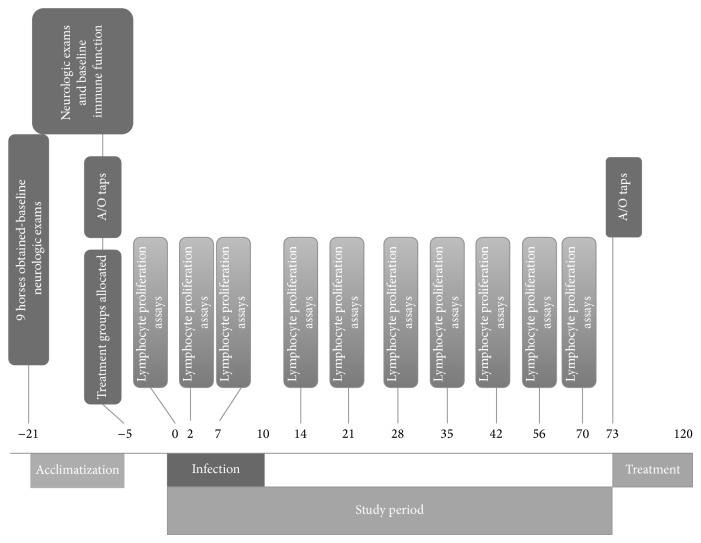
Experimental design for* S. neurona* infection study. Following a 2-week acclimation, nine horses had baseline neurologic examinations, immune function analysis, and CSF taps with samples submitted for SnSAG1 analysis. Horses were randomly assigned to either control or* S. neurona* experimentally infected treatment groups. Peripheral blood was collected on day −5, day −1, days 0–10, day 14, day 21, day 28, day 35, day 42, day 56, and day 70 for infection or immune function assays. CSF taps (A/O = atlanto-occipital space) were performed on day 5 and postinfection day 73.

Complete physical examinations were performed on all the horses upon arrival at Virginia-Maryland Regional College of Veterinary Medicine (VMRCVM). Baseline physical and neurologic exams were performed (Figure 1). All care and maintenance of the horses was in accordance with the guidelines established by the Animal Use and Care, IACUC committee at Virginia Tech, and the study was approved by the IACUC committee. All horses underwent a neurological/lameness examination two weeks prior to the start of the study. Investigators Lewis (RL) and Witonsky (SW) performed blinded neurologic exams separately for each horse including baseline scores. A 97-point scoring system was used to evaluate the horses' neurological/lameness status and is described in detail later in Table 4. The scoring system was based on a protocol established by Ellison et al. [18–20], which had been used for several previous United States Department of Agriculture (USDA) approved EPM studies. Briefly, the scores ranged from zero (normal) to 3 (severely affected) for 22 different parameters with a maximum total score of 97 (Table 4). The parameters measured included, but were not limited to, cranial nerve function, ataxia, weakness (paresis), and spasticity. The 22 parameters that were evaluated included dropping feed, tongue tone, apprehension of feed, drooling, lip, facial nerve and eyelid paresis, attitude, muscle atrophy, cauda equine, weakness, lameness, conscious proprioception based on crossing over of limbs, abduction of hind limbs, tripping, paresis, circling, pivoting and toe dragging of limbs, hypermetria, inconsistent placement, and tail pull. When a horse displayed a neurologic or lameness deficit, the appropriate score was assigned, and the horse was observed closely for two weeks prior to initiation of the study. If the horse's status was unchanged during this period, the animal was subjected to a more thorough neurologic evaluation (described in the following section) to minimize the possibility of a preexisting EPM infection. A few horses displayed gait and conformation abnormalities that resulted in a measurable elevated baseline neurologic/lameness score; however these abnormalities were determined to not be attributable to an EPM infection. We predicted that an* S. neurona* infection would alter the baseline score; thus horses with a preexisting minimal measureable baseline neurologic/lameness score remained in the study.

### 2.3. Serum and CSF Sample Collection (Day −5) prior to Infection

After a two-week acclimatization period (day −5), peripheral blood samples were collected, and horses were anesthetized. CSF was obtained from the atlanto-occipital (AO) joint space using a 3.5′′, 18 G spinal needle and submitted, along with the serum, to a commercial laboratoryfor SnSAG1 ELISA and to the laboratory at VMRCVM for routine cytology [19, 21]. CSF collection was also performed on postinfection day 73.

### 2.4. *S. neurona* Culture

Merozoites were obtained by a technique described by Ellison et al. [18, 20]. Briefly, SnSAG1 merozoites were isolated from CNS tissue from a horse previously diagnosed with EPM. These merozoites were then maintained in continuous culture. Merozoites were cultured in complete media (RPMI with L-glutamine (Mediatech, Herndon, VA), hepes buffer 25 mM (Mediatech, Herndon, VA), 2% heat inactivated fetal bovine serum (FBS) (Atlanta Biologics, Lawrenceville, GA), 50 IU/mL of penicillin/streptomycin solution (Sigma Chemical Company, St. Louis, MO), 1% sodium pyruvate solution (Mediatech, Herndon, VA)).

### 2.5. Horse Treatment Selection and Infection

Horses were randomly assigned to two treatment groups. As there is no sex bias with EPM, there was no selection for, or designation of, sex with a treatment group. Each group contained horses that had a baseline score of >0 after initial exam, for unrelated reasons, as previously discussed. On the day before infection (day −1), blood was collected from the left jugular vein of all horses into lithium heparinized blood collection tubes. Five horses were designated to receive the parasite in infected host leukocytes intravenously and constituted the experimentally infected group; the remaining four served as the noninfected control horses and received uninfected autologous lymphocytes. Briefly, 10 mls of peripheral blood collected from each horse in the infected group was cultured with 6000 live merozoites and incubated overnight at 37°C, 5% CO_2_. Peripheral blood from control horses was also incubated overnight without merozoites using identical conditions. Each day, the same amount of peripheral blood was obtained from each horse, and the previous day's (infected or control) autologous blood was administered. Horses were infected for 10 days. Blood was collected for specific assays on the following days: day 0, day 2, day 14, day 21, day 28, day 35, day 42, day 56, and day 70 [20].

### 2.6. Peripheral Blood Smear and Mononuclear Cell (PBMC) Isolation

Leukocyte differentials were obtained from direct blood smears made from each horse at each time point, prior to leukocyte enrichment. Slides were stained with modified Wright stain (Wright's Giemsa, Sigma). PBMCs were then isolated by density gradient centrifugation [22]. Whole blood samples were diluted with phosphate buffered saline (PBS) (Mediatech, Herndon, VA). Samples were enriched using Lymphoprep (Lymphoprep, Greiner, NJ). Cells were washed 2x's with PBS and enumerated.

### 2.7. Peripheral Blood Leukocyte Enumeration

Blood leukocyte cellularity was determined with a Multisizer 3 Coulter Cell Counter (Beckman Coulter, Fullerton, CA) using a sample volume of 10 *μ*L diluted with 10 mL of PBS. Cells (5 to 10 *μ*m diameter) were enumerated. Samples were diluted to a final concentration of 5 × 10^6^/mL in complete RPMI 1640 with L-glutamine, hepes buffer 25 mM, 10% heat inactivated FBS, penicillin (50 IU/mL), and streptomycin (50 IU/mL) [22].

### 2.8. Cytologic Analysis

Cytospins were performed on enriched peripheral blood leukocyte fractions to assess cell morphology, lymphocyte purity, and neutrophil contamination. Briefly, 50 *μ*L aliquots of cells (5 × 10^6^/mL) were combined with 150 *μ*L of PBS in a slide cytospin chamber and centrifuged for 5 min, 50 ×g, 23°C (Cyto-tek centrifuge, Sakura Finnetechnical Co, Tokyo, Japan). The slides were air dried, stained with modified Wright's stain, and enumerated by light microscopy. One hundred cells were counted across 10–15 fields [22].

### 2.9. Analysis of Peripheral Blood Leukocyte Marker Expression

A 100 *μ*L cell suspension (5 × 10^6^/mL) was stained with 50 *μ*L of the following diluted monoclonal antibodies (1 : 100 in PBS was added to individual wells of each sample): CD4^+^ (mouse anti-equine CD4^+^ antibody, 1 mg/mL, cell line HB61A IgG1, VMRD, Pullman, WA), CD8^+^ (mouse anti-equine CD8^+^ antibody, 1 mg/mL, cell line HT14A, IgG1, VMRD, Pullman, WA), B-cell (mouse anti-equine CD5^+^ antibody, 1 mg/mL, cell line B29A, IgG2a, VMRD, Pullman, WA), and DH59B/CD172a antibody (mouse anti-equine IgG1, 1 mg/mL, DH59B, VMRD, Pullman, WA) to stain monocytes and granulocytes (mouse anti-equine IgG1, 1 mg/mL, DH59B). Cells were incubated with the primary antibody for 20 min at 4°C. Cells were then washed with PBS, and 50 *μ*L of the secondary antibody was added to the wells. The secondary antibody added to the B-cell was PE rat anti-mouse IgG2a (for conjugation to B-cell antibody) (Pharmingen, San Diego, CA) at a dilution of 1 : 100. FITC rat anti-mouse IgG1 (Pharmingen, San Diego, CA) was used for conjugation to CD4, CD8, and DH59b antibodies, at a 1 : 100 dilution. Unstained control samples received PBS. The plate was washed, and cells were resuspended in 200 *μ*L of cold PBS for flow cytometric analysis. 7-AAD (Molecular Probes, Eugene, OR), if required in each step, was added at this time (see below). Cells were analyzed on an EPICS XL flow cytometer (Coulter, Hileah, FL). Samples were run until a minimum of 5000 (with an optimal count of 10,000) leukocytes or lymphocytes were captured (dependent on subset) as described below [12, 13, 22].

### 2.10. Analysis of Cell Viability and Apoptosis by 7-Amino Actinomycin D (7-AAD)

Following cell surface staining above, 1 *μ*g of 7-AAD in 200 *μ*L PBS was added to each sample. Plates were incubated for a maximum of 30 min on ice in the dark. Greater than 5,000 gated events per sample were collected by the EPICS XL flow cytometer. Based on the intensity of staining, cells were classified by their subset as 7AAD dull (live cells), 7AAD moderate (early apoptosis), and 7AAD bright (late apoptosis) [12, 13, 22].

In order to study the effect of PMA/I on apoptosis, additional cells were plated in three separate 96 well round bottom plates, and PMA/I was added (final concentration 20 ng/mL PMA, 10 pg/mL ionomycin) to each of one set of samples. Plates were incubated for 24, 48, and 72 hrs in a controlled environment (37°C, 5% CO_2_), with nonstimulated samples included as controls. At designated time points (24, 48, and 72 hrs), cell surface and 7-AAD staining of the cells were performed [12, 13, 22].

### 2.11. Carboxyfluorescein Succinimidyl Ester (CFSE) Staining

Aliquots of the cells (2 × 10^6^) were resuspended in 5 *μ*M of CFSE (Molecular Probes, Eugene, OR) for 10 min at 37°C [23]. Cells were then washed, plated, and stimulated with or without PMA/I for 72 hrs in a controlled environment (37°C, 5% CO_2_). Following incubation, primary and secondary antibodies were added as previously described. Flow cytometry was then performed: 5,000 or 10,000 cells gated events per sample. The number of cellular divisions for each subset was determined [23]. PMA/I-treated, merozoite-treated, and media only wells were included for each stain.

### 2.12. Live Merozoite Preparation

For assessment of antigen specific proliferation and apoptosis, merozoites were used for stimulating cells in some assays (i.e., lymphocyte proliferation assays, 7AAD, CFSE) [24]. Live* S. neurona* merozoites of the SN-37R strain were grown and maintained in African green monkey (*Cercopithecus aethiops*) kidney cells (CV-1 cells, ATTC CCL-70, American Type Culture Collection, Rockville, MD, USA). The* S. neurona* merozoites were harvested from CV-1 cells by removing the complete media (RPMI with L-glutamine, hepes buffer 25 mM, 2% heat inactivated FBS, 50 IU/mL of penicillin/streptomycin solution, 1% sodium pyruvate solution), including the merozoites. The suspension was filtered through a 3 *μ*M filter and spun at 1500 rpm (350 ×g) for 10 min at room temperature and resuspended in fresh complete media. The merozoites were enumerated with a hemocytometer and then resuspended to 1 × 10^5^/mL with complete media containing 10% heat inactivated FBS. Merozoites (100 *μ*L/well) were then incubated with the equine leukocytes [24].

### 2.13. Lymphocyte Proliferation Assays

Plates containing 100 *μ*L aliquots of each horse's enriched lymphocytes (2 × 10^6^/mL) in triplicate wells were cultured together with 100 *μ*L of the appropriate mitogen or live merozoites (1 × 10^5^/mL), or media only [1, 12, 13, 22]. Final concentrations of mitogens in the wells were 5 *μ*g/mL ConA (Sigma Chemical Company, St. Louis, MO), 1 *μ*g/mL PWM (Sigma Chemical Company, St. Louis, MO), 20 ng/mL PMA (Sigma Chemical Company, St. Louis, MO), and 10 pg/mL ionomycin (Sigma Chemical Company, St. Louis, MO). Cell spontaneous proliferation was assessed with cells cultured in triplicate wells containing complete media (200 *μ*L/well). Cells were incubated for 48 hrs at 37°C in humidified 5% CO_2_ and then pulsed with 1 *μ*Ci^3^H-thymidine. Plates were harvested 18–24 hrs later using a Filtermake Harvester (Packard Bioscience, Billerica, MA). Stimulation indices were calculated for each mitogen by dividing the mean counts per minute (CPM) of wells with mitogen by the mean CPM of the unstimulated cells in media, which represented spontaneous proliferation [12, 13, 22].

### 2.14. EPM Treatment Poststudy

Horses in the merozoite-infected group were treated with trimethoprim/sulphadiazine (20 mg/kg sulphadiazine) and pyrimethamine (1 mg/kg) for a minimum of 12 weeks, or until they returned to their preinfected neurologic status, and the SnSAG1 ELISA returned to within normal reference range. Upon completion of the study, all the horses were placed in suitable homes through adoption.

### 2.15. Statistics and Data Analysis

Horses 3, 5, 6, 7, and 9 received ten doses of the protozoan in autologous lymphocytes via intravenous injection and constituted the experimentally* S. neurona*-infected group. Horses 1, 2, 4, and 8 served as controls. Horse 2 received 1 dose of* S. neurona* in error and was removed from the study. Data from horse 2 was not included as either an infected or noninfected animal.

Standard residual plots were used to assess model adequacy, and logarithmic values were also analyzed for many data sets in order to stabilize the variance between individual data points. Normal probability plots showed that logarithmic values lay closer to a straight line.

Since there were more than two treatments to be assessed in this study, analysis of variance was conducted using the glimmix procedure of the SAS system (version 9.2) (SAS Institute Inc, Cary, NC). This procedure was utilized for most of the individual analyses performed, including results of flow cytometry, neurologic scoring over time, and lymphocyte proliferation assays. As there were dramatically fewer time points available for CD8^+^ cells in late apoptosis (i.e., not all time points contained sufficient cell numbers in late apoptosis to accurately analyze the data), an Exact Wilcoxon two-sample test was performed on CD8^+^ cells in late apoptosis.

Results were expressed as an adjusted *P* value and were referenced against the mean value for each group ± standard deviation. The actual number of horses used to generate the results was at times different for select tests, due to limited data collection of some samples, or reduced cellular survival in these individuals. Significance was set at *P* < 0.05.

## 3. Results

### 3.1. Complete Blood Count and Differentials

All CBC and blood cytologic profiles for the 9 horses were within acceptable reference intervals prior to initiation of the study (data not shown).

### 3.2. Serum and CSF Analysis

All horses had serum titers of <2, except for horse number 6, which had a titer of 4 at day 5. A titer ≥32 is considered significant in cases with accompanying neurologic signs, including in experimentally induced cases with neurologic signs that received 6000 organisms/day for 10–14 days (Table 1). For this study, horses were only infected for 10 days. By day 73 after inoculation, all* S. neurona*-infected horses had serum titers ≥32, consistent with active infection.* S. neurona*-infected horses 3, 5, and 7 had positive CSF titers, which were ≥1. Serum from horses 1 and 8 had titers that were ≥2.

### 3.3. CSF Cytology

Several horses had increased red blood cell (RBC) levels, which were attributed to iatrogenic contamination (Table 2). We believe that this did not adversely affect the SnSAG1 ELISA results as all horses had a serum titer of <2 prior to the initiation of the experiment.

### 3.4. Neurologic Examination Scores

**Figure 2:**
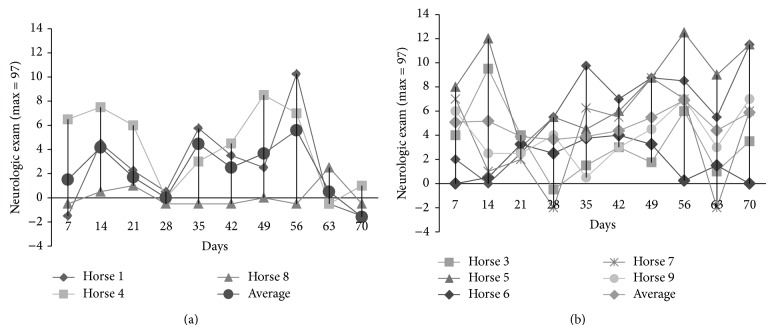
Neurologic scores by (a) individual total non-experimentally infected neurologic score control (top) and (b)* S. neurona* experimentally infected (bottom) horses. All horses were scored biweekly via a comprehensive neurologic panel. Each parameter was assessed a score from 0 (normal) to 3 (very abnormal), with a maximum of 97. (a) Total neurologic score is reported. Baseline = preacclimatization score. Week −1 = score following acclimatization, but prior to infection. Week 1 = first week of infection with* S. neurona*. Results are reported by baseline-individual horse response by day and the average response of the horses by treatment by day.

**Figure 3:**
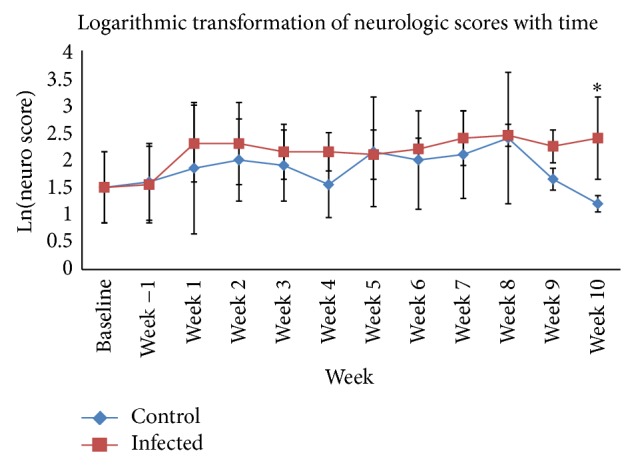
Log-transformed neurologic scores (all parameters) over time. Horses were scored biweekly for a multitude of different parameters in order to detect neurologic signs. The average neurologic score was log-transformed and plotted against experimental time to equalize variance between samples. Error bars represent one standard deviation about the mean. Baseline = preacclimatization score. Week −1 = score following acclimatization, but prior to infection. Week 1 = first week of infection with* S. neurona*. Error bars represent one standard deviation about the mean.

While all 5* S. neurona*-inoculated horses exhibited neurologic signs and had an increased neurologic score over the course of the study, horses varied as to when their neurologic deficits were most significant (Figure 2). Initial neurologic signs were noted on day 5 of infection and on day 10 after infection. All* S. neurona* challenged horses displayed progressive clinical disease with moderate to severe neurologic signs (Figure 3). The neurologic signs included, but were not limited to, cranial nerve deficits, changes in attitude, and ataxia. The level of neurologic deficits was higher than anticipated, and as a result, the decision to stop the infection process on day 10 was made based on genuine concern for the health of the horses. According to Ellison (via phone communication), horses already displaying this level of neurologic deficits would become progressively worse from this time point, and it was therefore anticipated that these horses would progress to a Grade II status by day 60 after infection (the standard level of ataxia induced with this dose and model) [18–20]. Neurologic scores for horse 3 peaked within the first 14 days after infection, and horses 5, 6, 7, and 9 peaked between days 42–56 of the experiment. All experimentally* S. neurona*-infected, except horse 7, and non-experimentally infected control horses, except horse 8, had a higher score on day 56. All* S. neurona*-infected horses demonstrated a detectable level of ataxia compared to control horses at days 7, 21, and 70 (data not shown) Experimentally infected horses exhibited a peak in cranial nerve signs prior to the peak in ataxia, the latter of which was more pronounced towards the end of the study period. Logarithmic values were used in the statistical analysis in an attempt to stabilize the variance and as these were closer to a straight line on the normal probability plots. A significant difference (*P* < 0.05) between the control and infected population's mean overall neurologic score was present on day 70. A trend towards a difference (0.05 < *P* < 0.1) was seen at days 7 and 28.

### 3.5. Enriched Peripheral Blood Leukocyte Cytology

Cytologic examination of the enriched peripheral blood leukocytes was not remarkable across treatment or time (data not shown). The average differential count obtained from these enriched cell suspensions was 6% neutrophils, 87.5% lymphocytes, and 6.5% monocytes.

### 3.6. Lymphocyte Proliferation

**Figure 4:**
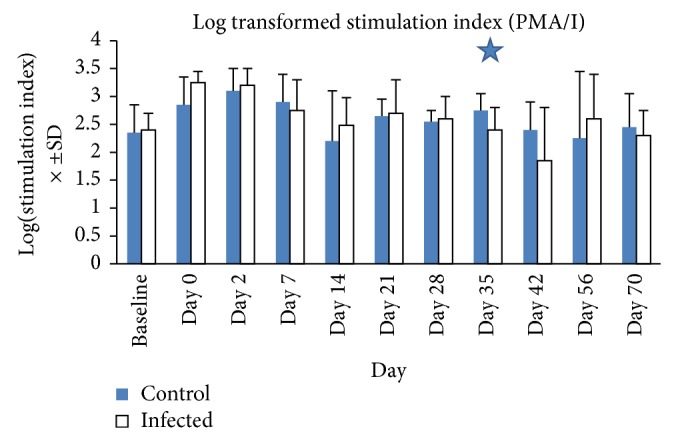
Differences in log-transformed stimulation indices for incubation with PMA/I between* S. neurona* experimentally infected and control horses. Control horses had a significantly higher stimulation index for PMA/I responses on Day 35, with a pattern demonstrating higher indices in the control group towards the end of the experiment. Lymphocyte proliferation assays using the (^3^H)-thymidine assay were performed with a variety of mitogens, including the pan-leukocyte stimulant, PMA/I. Stimulation indices were calculated by dividing the average counts per minute for cells stimulated with each mitogen, by the counts per minute for spontaneously proliferating cells. Average stimulation index was log-transformed and plotted against time for control (blue) and infected (white) horses. The error bars represent standard deviation about the mean. The blue star indicates a significant difference (*P* < 0.05). Day 0 = first day of experiment (Day 1 of infection).

**Figure 5:**
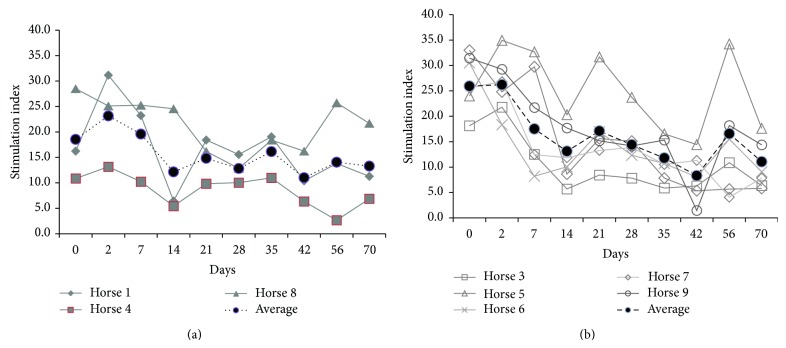
Individual PMA/I stimulation indices for non-experimentally infected (a) and* S. neurona* experimentally infected (b) horses. Lymphocyte proliferation assays using the (^3^H)-thymidine assay were performed with a variety of mitogens, including the pan-leukocyte stimulant, PMA/I. Stimulation indices were calculated by dividing the average counts per minute for cells stimulated with each mitogen, by the counts per minute for spontaneously proliferating cells. Results are reported by individual horse response and the average response of the horses by treatment by day.

Enriched peripheral blood lymphocytes from* S. neurona*-infected horses cultured with PMA/I demonstrated a significantly lower stimulation index compared to controls on day 35 (Figures 4 and 5). No consistent differences were observed in proliferation response of* S. neurona*-infected cells to ConA (T-cell), PWM (B-cell), or antigen specific- (merozoites-) induced proliferation as detected by (^3^H)-thymidine incorporation, throughout the study (data not shown).

### 3.7. Peripheral Blood Leukocyte Cell Surface Marker Expression

Cell surface marker expression of fresh and cultured leukocytes, in general, did not differ between the two treatment groups, with the exception of neutrophils. The* S. neurona*-infected group had a significantly higher percentage of neutrophils at days 0 and 14 (data not shown).

### 3.8. Apoptotic Changes in Immune Subsets

**Figure 6:**
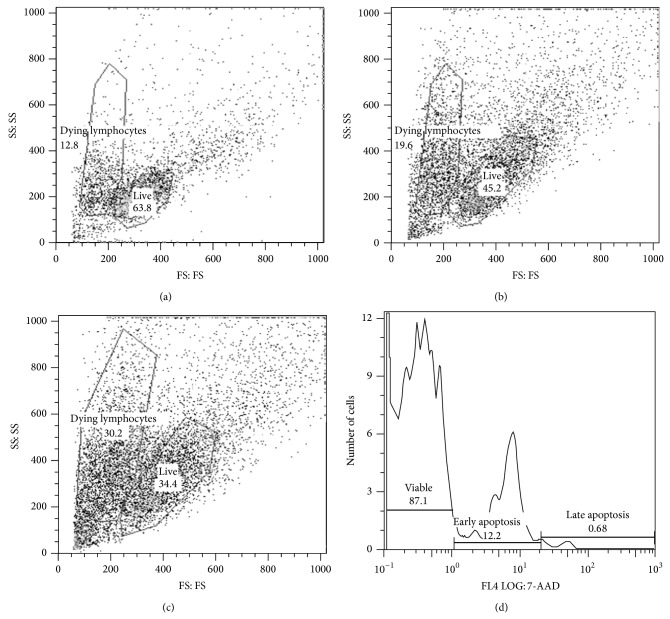
Gating for apoptosis data. 2a: gate of live versus dying lymphocytes after (a) 24 hrs, (b) 48 hrs, and (c) 72 hrs. (d) A sample of the 7-AAD gating of viable, early, and late apoptotic cells.

For cells cultured with media only, PMA/I, and merozoite stimulated samples, viable, early, and late apoptotic populations were defined based on 7-AAD staining (Figure 6). All samples were analyzed using the same electronic gates. Most data are presented in the context of significant differences between the experimentally infected versus non-experimentally infected control animals (Table 5).

In cells cultured with media only, the percentage of CD8^+^ cells undergoing apoptosis tended to be higher in* S. neurona*-infected horses. In PMA/I cultured wells, neutrophils (PMN) had decreased apoptosis (day 14) and viability (days 35 and 42) (data not shown). Monocytes (mono) had decreased apoptosis (days 21, 28, and 35) and decreased viable cells at (days 42 and 56). CD4^+^ and CD8^+^ cells had increased viability at days 7 and 70; CD8^+^ cells had significantly increased apoptosis present at day 42. In contrast, B cells had increased viability on days 21 and 28. In cells cultured with* merozoites,* there were individual treatment groups with increased apoptosis at select time points, but no consistent changes across time (data not shown). Apoptosis was increased in CD4^+^ cells (days 35 and 70) and CD8^+^ cells (days 42 and 70).

### 3.9. Alterations in Immune Populations Based on CFSE Proliferation

**Figure 7:**
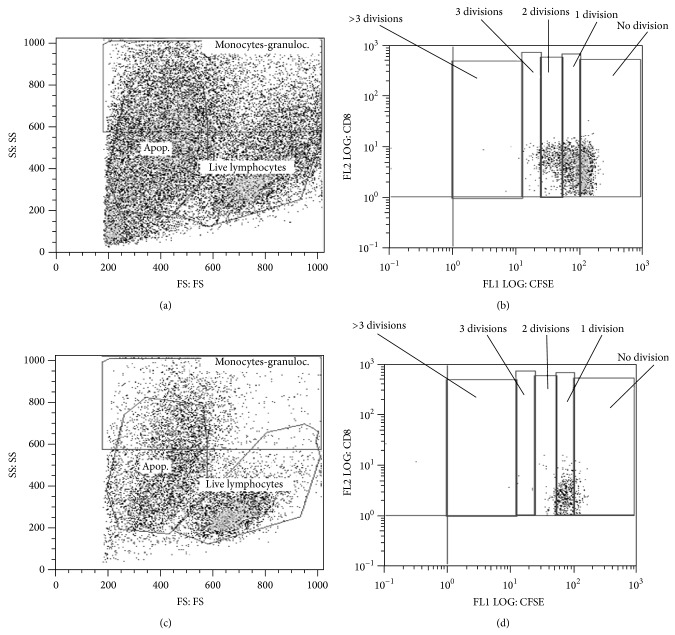
Effects of PMA/I and merozoite stimulation on peripheral leukocyte proliferation as detected using CFSE. Lymphocytes from control and* S. neurona*-infect horses were incubated with CFSE and cultured with (a) PMA/I or (b) unstimulated for 72 hrs at 37°C, 5% CO_2_. Cells were washed and stained for leukocyte markers, and proliferation based on number of divisions was determined, (c) PMA/I stimulated, and (d) unstimulated.

Samples were gated on live versus dying/apoptotic gates, and then cell divisions were marked (Figure 7, Table 3) [23]. For each time point, stimulation, and immune cell subset, data were gated and statistically analyzed based on quantitative differences in number of proliferations present per sample. The significant differences are presented in Table 3 to provide the pattern of response. CD4^+^ cells (days 2, 7, 21, and 56) and CD8^+^ cells (days 2, 21, and 28) from* S. neurona*-infected horses had increased numbers of divisions/proliferation when stimulated with PMA/I. When cultured with live merozoites, CD4^+^ cells from the* S. neurona*-infected horses had decreased cell divisions/proliferation on day 28, but increased divisions/proliferation on day 42. CD8^+^ cells had decreased divisions/proliferation in infected horses at days 28, 56, and 70, suggesting a limited ability of leukocytes to respond to merozoites. Due to a consistently limited cell sample recovery after 72 hrs of culture, the B cells and monocytes were not evaluated.

## 4. Discussion

Studying EPM in horses is difficult due to the challenges of developing a reliable infection model. In the present study, we were able to validate Ellison's model of infection, by showing that experimentally* S. neurona*-infected horses developed neurologic clinical signs as well as generating positive serum and CSF titers. Using this model, we made several noteworthy observations regarding the equine immune response to active* S. neurona* infection. We observed mitogen-induced differences in leukocyte apoptosis and proliferation during infection and after infection. Of interest, our data suggest that* S. neurona* infection alters the ability of antigen presenting cells to stimulate CD4^+^ and CD8^+^ proliferation, which will be the subject of future studies.

All* S. neurona* infected horses had increased serum and CSF antibody levels after infection. Horses with SnSAG1 CSF titers ≥1 indicated intrathecal production of antibodies and thus active infection. The lowest detection titer for SnSAG1 CSF as performed by Pathogenes was changed by the company during our study period. Prior to the experiment, the first dilution performed was 1 : 2. Therefore the lowest negative titer for the test was <2. During the time in which we performed our experiment (thus applicable to postexperiment CSF SnSAG1 results), Pathogenes lowered the negative titer to <1. This perceived reduced sensitivity in our prechallenge samples unlikely impacted our data, as all horses had negative serum titers at this time of collection. Based on a postchallenge positive serum titer, it appeared that control horses 1 and 8 may have had a natural exposure to* S. neurona*. Additionally, based on control horse 4 having a positive CSF titer, this suggests natural exposure in which* S. neurona* was sequestered from the immune response until entering the CNS. Throughout the study, all the horses were kept together in a fenced pasture, and while the hay was kept in a covered barn prior to feeding, the potential for natural exposure was possible. When the study was performed, ELISAs to determine the antibodies to different SAGs were not available, and unfortunately the sera from these horses are no longer available. Thus, we were unable to determine if the “naturally exposed” horses were infected by a non-SAG-1 expressing strain. However, these two control horses never developed positive CSF titers. It is possible that natural exposure to* S. neurona* could have affected the immune responses of these control horses and thus the relative differences between experimentally infected and non-experimentally infected horses required careful review of the data. However, we do believe that the significant findings of the* S. neurona* experimentally infected horses, compared to the control horses, even with possible natural exposure of some, are still valid. For most cases, we predict that the findings would have been even more dramatic without possible natural exposure.

With regard to the merozoite-infected leukocyte model developed by Ellison et al., we were able to confirm successful infection and manifestation of clinical EPM [18–20]. However, we only infected the horses for 10 days, based on the severity of clinical neurologic signs. According to Dr. Ellison (personal communication), these signs were more severe than typical of Day 10. If the infection process had been allowed to continue for 14 days, we predicted that the clinical signs, and possibly the immune responses, would have been more pronounced. Our primary rationale for discontinuing the experimental infection protocol at day 10 was for the safety of the horses.

The utilization of the different mitogens (ConA, PWM, PMA/I) in culture allowed us to assess the T cell, B cell, and pan-leukocyte response, in these horses during active* S. neurona*-induced infection. With PMA/I-induced proliferation, we observed a significant decrease in proliferative response of infected horses' lymphocytes at day 35. This was in contrast to previous studies, where the decreased proliferation response was present at multiple time points [12, 13]. However, we did observe a pattern of declining proliferation late in the infection stage. Possible explanations for the lack of additional differences would be (a) differences in the stage/severity of infection in the facts that these horses were infected with a lower total dose compared to other studies with experimentally infected horses and that (b) some of the control horses were natural exposed to* S. neurona* and developed similar decreased PMA/I responses, thus masking the decrease in PMA/I response of experimentally infected horses. The lack of more consistent significant differences in immune cell subsets and proliferation could be due to individual variability among horses with different genetic backgrounds, possible natural exposure, and dose of infection used, as well as individual differences in immune responses. Additionally, it is possible that the* S. neurona* infection could have caused transient changes in the immune response, based on the dynamic nature of the immune response (i.e., development of protective response versus development of disease). Therefore, we focused on differences when an overall trend was seen, even if there were just a few significant differences.

Analysis of the apoptosis and CFSE results revealed that there were multiple time points in which unstimulated CD8 cells (days 0, 14, 35, and 42) from experimentally infected horses had increased apoptosis. With regard to PMA/I stimulation, neutrophils had decreased viability at days 35 and 42; monocytes had decreased apoptosis at days 21, 28, 42, and 56. B-cells had increased viable cells at days 21 and 28 and decreased viable cells at day 35, which may have adversely impacted the observed decrease in PMA/I-induced proliferation. Based on the CFSE experiment, PMA/I stimulation demonstrated increased proliferation in CD4 (days 2, 7, 21, 56, and 70) and CD8 cells (days 2, 21, and 28), whereas cells stimulated with merozoites resulted in changes in CD4 (decreased day 28, increased day 42) and decreased CD8 proliferation (days 28, 56, and 70). With regard to determining whether suppressed PMA/I proliferation was due to increased apoptosis or decreased proliferation, as only day 35 was statistically significant, our interpretation of the possible mechanisms is very limited. In examining the day 35 data, there is a spontaneous increase in CD8 apoptosis, and with PMA/I stimulation, there is a decrease in viable neutrophils and B-cells. Based on these data, plausible mechanisms for the decreased PMA/I induced response would be decreased PMN, B-cell, and CD8 viability, with increased apoptosis of the CD8 cells, and will require further investigation.

In regard to the changes in immune response during active* S. neurona* infection, the observations of increased PMA/I-induced proliferation with CFSE and decreased proliferation to merozoites in the* S. neurona*-infected horses suggest that both CD4^+^ and CD8^+^ cells were at a heightened stage of activation to nonspecific stimuli (i.e., PMA/I). With merozoite stimulation,* S. neurona*-infected horses had decreased cellular proliferation. This decreased response could possibly be associated with an impaired ability of antigen presenting cells (APC) to process and present* S. neurona* antigen to CD4^+^ and CD8^+^ cells. Merozoites must be processed and presented by antigen presenting cells (APC), which is a time dependent process, in order to stimulate T-cells. It is also possible that* S. neurona* impairs the ability of antigen presenting cells to present novel antigen to the CD4^+^ and CD8^+^ cells. We do not know if this is specific to* S. neurona* or any antigen. Another possibility is that the CD4^+^ and CD8^+^ cells had a decreased ability to respond to* S. neurona* (T-cell specific response) due to a loss in secondary signals. Alternatively, CD4^+^ and CD8^+^ cells from* S. neurona*-infected horses could be more susceptible to the merozoites. This would result in decreased CFSE-mediated proliferation of merozoite-infected cells from infected versus control horses.

In this study, we were not able to distinguish a unique and consistent change in equine immune response to* S. neurona* infection and relate it to susceptibility. Decreased viability of neutrophil populations was noted and likely was due to active infection. The observed decrease in monocyte apoptosis could be an effect of active infection or a direct inhibitory effect of* S. neurona* on monocyte function (as demonstrated with other protozoan) [25]. Likewise, observed increased B cell viability could also be associated with response to active infection and will be evaluated in future studies. Additional studies are needed to further define these mechanisms.

To summarize, the results from this study demonstrate that horses experimentally infected with* S. neurona* develop clinical disease and generate antibodies to* S. neurona* in the serum and CSF. These* S. neurona*-infected horses had altered immune cell subset expression, which changed during disease progression. Furthermore, the functionality of these immune cells as measured by proliferation, as well as viability and degree of cell death, was also intermittently affected. Most interestingly,* S. neurona*-infected horses had decreased antigen specific proliferation responses compared to non-experimentally infected horses; however, non-antigen specific responses (i.e., mitogen-induced) were not decreased throughout most of the study. These data suggest that the process between antigen presenting cell (monocyte/dendritic cell) and/or T cell antigen recognition may be impaired in* S. neurona*-infected horses. The nature of this defect in the horse's immune response could possibly be associated with the particular stage of development of clinical EPM, following exposure to* S. neurona*, but needs to be investigated further under conditions in which the infection duration is longer. Still, these preliminary experiments raise interesting new questions regarding the precise immune response of cellular subsets to infection with* S. neurona*.

## Figures and Tables

**Table 1:** SnSAG1 serum and CSF titers for all horses, before and after experimental infection.

Horse	Infection status	Initial serum SnSAG1 titer	Initial CSF SnSAG1 titer	Postexperiment serum SnSAG1 titer	Postexperiment CSF SnSAG1 titer
1	Control	<2	<2	32	<1
2	Removed from study	<2	<2	16	1
3	*S. neurona*-infected	<2	<2	80	2
4	Control	<2	<2	<2	2
5	*S. neurona*-infected	<2	<2	32	2
6	*S. neurona*-infected	4	<2	32	<1
7	*S. neurona*-infected	<2	<2	80	1
8	Control	<2	<2	32	<1
9	*S. neurona*-infected	<2	<2	32	<1

Serum and CSF SnSAG-1 analysis was performed prior to and following the conclusion of the experiment (after Day 73). Infection status is marked as control or *S. neurona*-infected. Horse 2 received a dose of the parasite in error and was excluded from statistical analysis.

**Table 2:** Baseline CSF cytology data and SAG1 titer on Day −5.

Horse	Infection status	Color	Transparency	WBC/uL	RBC/uL	Glucose (mg/dL)	Protein (mg/dL)	SnSAG1 ELISA titer
1	Control	Colorless	Clear	1	1575	49.1	62.4	<2
2	Removed from study	Colorless	Clear	2	158	50.2	59.1	<2
3	*S. neurona*-infected	Colorless	Clear	0	1	49.3	45.1	<2
4	Control	Colorless	Clear	1	52	50.9	28.2	<2
5	*S. neurona*-infected	Colorless	Clear	1	0	48.6	73.8	<2
6	*S. neurona*-infected	Colorless	Clear	2	11	51.3	42.0	<2
7	*S. neurona*-infected	Colorless	Clear	1	0	50.8	33.4	<2
8	Control	Colorless	Clear	0	0	52.2	37.1	<2
9	*S. neurona*-infected	Colorless	Clear	0	0	56.5	55.7	<2

Results of cytologic analysis of the preexperiment CSF sample, including SAG-1 titer, are shown. Results presented include infection status, color, transparency, white blood cell (WBC) and red blood cell (RBC) count, glucose, protein, and SnSAG titer. Horse 2 received a dose of the parasite in error and was excluded from statistical analysis.

**(a):** 

PMA	Percent CD4	Percent CD4 0 divisions	Percent CD4 1 division	Percent CD4 2 divisions	Percent CD4 3 divisions	Percent CD4 >3 divisions
Day 2 CD4 Control	35.8 ± 4.68	52 ± 5.67	39.23 ± 5.65^*^	11.47 ± 0.86^*^	1.32 ± 0.32^*^	0.19 ± 0.09
Day 2 CD4 Infected	43.74 ± 4.37	33.44 ± 7.08	49.8 ± 4.86	18.86 ± 2.15	2.892 ± 0.59	0.442 ± 0.18
Day 28 CD4 Control	34.7 ± 0.81	51.2 ± 13.02	46.63 ± 13	3.22 ± 0.90^*^	1.62 ± 0.29^*^	2.50 ± 0.08^*^
Day 28 CD4 Infected	33.04 ± 3.79	50.64 ± 6.7	50.12 ± 6.49	2.114 ± 0.52	0.424 ± 0.11	1.82 ± 0.37

**(b):** 

PMA/I	Percent CD8	Percent CD8 0 divisions	Percent CD8 1 division	Percent CD8 2 divisions	Percent CD8 3 divisions	Percent CD8 >3 divisions

Day 2 CD8 Control	10.49 ± 1.3	54.27 ± 2.39^*^	38.4 ± 3.7^*^	9.40 ± 1.01^*^	1.44 ± 1.56^*^	0.44 ± 0.08
Day 2 CD8 Infected	10.05 ± 1.16	36.78 ± 7.97	44.62 ± 4.54	20.7 ± 3.26	2.948 ± 2.91	0.636 ± 0.12
*Merozoites *						
Day 28 CD8 Control	26.55 ± 0.65	29.26 ± 2.36^*^	63.05 ± 2.26^*^	10.11 ± 0.8^*^	1.185 ± 0.26^*^	1.23 ± 0.01
Day 28 CD8 Infected	23.32 ± 1.88	18.78 ± 1.69	76.22 ± 1.03	6.41 ± 0.77	0.88 ± 0.26	1.89 ± 0.53
Day 56 CD8 Control	17.63 ± 2.26	65.57 ± 23.2^*^	28.46 ± 16.61^*^	7.28 ± 6.36^*^	0.12 ± 0.09	0 ± 0
Day 56 CD8 Infected	19.52 ± 3.17	81.72 ± 10.61	19.14 ± 10.87	1.16 ± 0.74	0.12 ± 0.02	0.13 ± 0.08
Day 70 CD8 Control	23.1 ± 2.04	64.67 ± 14.5^*^	37.53 ± 14.16^*^	1.78 ± 1.09^*^	0.09 ± 0.09^*^	0.14 ± 0.07^*^
Day 70 CD8 Infected	21.06 ± 2.5	82.62 ± 5.57	19.75 ± 6.27	0.62 ± 0.13	0.12 ± 0.06	0.04 ± 0

Lymphocytes from control and *S. neurona*-infected horses were stained with CFSE and then cultured with no stimulation, PMA/I, or live merozoites for 72 hr at 37°C, 5% CO_2_. Samples were then stained for CD4 (a) or CD8 (b) and the number of divisions per sample was determined. The average number of cells per division ± SEM was determined for control and infected horses. Statistical differences are marked as ^*^ *P* < 0.05.

**Table 4:** Neurologic scoring sheet for observations of clinical parameters (total possible 97).

Category	Action	Description	Number	Description 2
Eating	Drops feed	<1/4 lb	1	(max = 3)
		≥1/4 lb ≤ 1/2 lb	2	
		>1/2 lb	3	
	Tongue tone decreased	Normal mastication	1	(max = 3)
		Abnormal mastication	2	
		Paresis	3	
	Abnormal feed prehension	Normal	0	(max = 2)
		Unable to masticate	1	
		Bites food	2	

Head	Drooling	Does not drool	0	(max = 2)
		When eating	1	
		Continuously	2	
	Lip paresis	Normal	0	(max = 3)
		Perceptible when eating	1	
		Perceptible continuously	2	
		Lip hangs	3	
	Facial nerve paresis	Normal	0	(max = 3)
		Perceptible	1	
		Moderate	2	
		Severe	3	
	Eyelid paresis	Normal	0	(max = 3)
		Ventrally away from eye	1	
		Over 1/4 of eye	2	
		With corneal lesion	3	
	Attitude	Normal	0	(max = 3)
		Depressed	1	
		Aggressive	2	
		Somnolent	3	

Muscle	Muscle atrophy	Normal	0	(max = 3)
		Just perceptible	1	
		Immediately noticeable	2	
		Severe atrophy	3	

Rear end	Cauda equina	Normal	0	(max = 3)
		Holds tail rigid	1	
		Dribbling urine	2	
		Rectum paretic	3	

Movement	Weakness	Normal	0	Left fore (LF), right fore (RF), left hind (LH), right hind (RH) (max = 12)
		While walking	1	
		Markedly reduced strength	2	
		Can pull horse over easily/recumbent	3	
	Lameness	None seen	0	(max = 3)
		Just noticed at walk	1	
		Head bob or hip drop at walk	2	
		Reluctant to use limb	3	

Conscious proprioception	Crossing over limbs	Normal	0	LF, RF, LH, RH (max = 12)
		Slightly slow to place limb	1	
		back to normal position		
		No resistance to abnormal placement of limb	2	
		Horse unaware of limb position and unable to correctly place limb	3	
	Abduction of hind limbs	Normal	0	LH, RH (max = 6)
		Slightly slow to place limb back to normal position	1	
		No resistance to abnormal placement of limb	2	
		Horse unaware of limb position and unable to correctly place limb	3	
	Tripping	None seen	0	(max = 3)
		Sometimes	1	
		Often	2	
		Often and falls to knees	3	

Limbs	Paresis	Normal	0	(max = 3)
		Detectable at normal gaits but exacerbated by manipulative actions	1	
		Obvious at normal gaits or postures	2	
		Very prominent at normal gait	3	
	Circling	Normal	0	Counterclockwise, clockwise (max = 6)
		Occasional abnormal limb placement	1	
		Does not move normally in circle	2	
		Refuses to circle/falls	3	
	Pivoting	No pivoting seen	0	Counterclockwise, clockwise (max = 6)
		Pivots occasionally on inside hind limb	1	
		Pivots frequently	2	
		Will not pick up hind leg-pivots continuously	3	
	Toe dragging	No toe dragging seen	0	Left, Right (max = 6)
		Occasionally drags a hind limb	1	
		Frequently drags hind limb	2	
		Constantly drags hind limbs	3	

Backing up	Hypermetria	Normal movement	0	(max = 3)
		Slightly hypermetric in one limb	1	
		Moderately hypermetric in one limb/slight hypermetria in both hind limbs	2	
		Very obvious hypermetria	3	
	Inconsistent placement	Normal placement	0	(max = 3)
		Slightly wide based on placement of hind limbs	1	
		Wide based on hind limb placement	2	
		Places limbs very abnormally (touching, very wide), almost falls	3	

Tail pull	At the walk	Normal	0	Left, Right (max = 6)
		Slight lack of resistance to tail pull	1	
		Easily pull horse off track	2	
		Horse almost falls when tail pulled	3	

Maximum total possible				**97**

Horses were examined biweekly and scored based on a variety of different parameters, listed above. For most categories, a score of zero represented a normal response to evaluation of this parameter, and a score of three was the maximum neurologic score possible. The scoring system was based on that used in previous experiments by investigators using the intravenous method of infection.

**(a):** 

Immune cell	Stimulation	Gate	Day	Time (hrs)	Change
CD4	PMA/I	Viable	7	24	Increase late apop
				72	Increase viable
CD4	PMA/I	Apop/dying	7	24	Decrease late apop
			14	24	Decrease late apop
CD8	PMA/I	Viable	7	48	Increase viable
			42	48	Increase early apop
			70	48	Increase viable
				72	Decrease early apop
Neutrophils	PMA/I	Viable	14	72	Decrease early apop
			35	24	Decrease viable
				48	Decrease late apop
			42	48	Decrease viable
Monocytes	PMA/I	Viable	21	48	Decrease late apop
			28	24	Decrease late apop
			35	24	Decrease late apop
				48	Increase early apop
				72	Increase late apop
			42	48	Decrease viable
		Viable	56	24	Decrease viable
			70	48	Increase late apop
			70	72	Increase late apop
B-cells	PMA/I	Viable	0	24	Increase late apop
			2	24	Increase late apop
			14	48	Decrease viable
			21	24	Increase viable
				72	Increase early apop
			28	24	Increase viable
			35	48	Increase late apop
			70	24	Increase early apop
		Apop/dying	21	24	Increase early apop
			35	72	Decrease viable
			70	24	Decrease late apop

**(b):** 

Immune cell	Stimulation	Gate	Day	Time (hrs)	Change
CD4	Merozoite	Viable	35	24	Increase late apop
			70	24	Increase late apop
		Apop/dying	70	24	Increase viable
				24	Decrease early and late apop
				72	Decrease viable, increase apop
CD8	Merozoite		42	24	Increase early apop
			70	72	Increase late apop
Neutrophils	Merozoite		28	24	Decrease early apop
			56	24	Increase viable
		Viable	70		Increase viable early
					Later increase early and late apop
				72	Increase early apop
Monocytes	Merozoite		56	24	Increase viable control
			70	24	Increase late apop
B-cells	Merozoite	Live	0	24	Increase late apop
			2	48	Increase late apop
			14	48	Decrease viable
			21	24	Increase viable
				72	Increase early apop
			28	24	Increase viable
			35	48	Increase late apop
			70	24	Increase early apop
		Dying	35	72	Increase viable
			56	72	Decrease viable
			70	24	Decrease viable
				72	Increase early apop

The immune cell subset, treatment, viable/dying gate, day, time point and significant change are reported.
